# Interplay of Nutrition and Psychoneuroendocrineimmune Modulation: Relevance for COVID-19 in BRICS Nations

**DOI:** 10.3389/fmicb.2021.769884

**Published:** 2021-12-17

**Authors:** Arundhati Mehta, Yashwant Kumar Ratre, Krishna Sharma, Vivek Kumar Soni, Atul Kumar Tiwari, Rajat Pratap Singh, Mrigendra Kumar Dwivedi, Vikas Chandra, Santosh Kumar Prajapati, Dhananjay Shukla, Naveen Kumar Vishvakarma

**Affiliations:** ^1^Department of Biotechnology, Guru Ghasidas Vishwavidyalaya, Bilaspur, India; ^2^Department of Psychology, Government Bilasa Girls Post Graduate Autonomous College, Bilaspur, India; ^3^Department of Zoology, Bhanwar Singh Porte Government Science College, Pendra, India; ^4^Department of Biochemistry, Government Nagarjuna Post Graduate College of Science, Raipur, India; ^5^Department of Botany, Guru Ghasidas Vishwavidyalaya, Bilaspur, India

**Keywords:** BRICS, COVID-19, immunity, nutrition, neuropsychology

## Abstract

The consequences of COVID-19 are not limited to physical health deterioration; the impact on neuropsychological well-being is also substantially reported. The inter-regulation of physical health and psychological well-being through the psychoneuroendocrineimmune (PNEI) axis has enduring consequences in susceptibility, treatment outcome as well as recuperation. The pandemic effects are upsetting the lifestyle, social interaction, and financial security; and also pose a threat through perceived fear. These consequences of COVID-19 also influence the PNEI system and wreck the prognosis. The nutritional status of individuals is also reported to have a determinative role in COVID-19 severity and convalescence. In addition to energetic demand, diet also provides precursor substances [amino acids (AAs), vitamins, etc.] for regulators of the PNEI axis such as neurotransmitters (NTs) and immunomodulators. Moreover, exaggerated immune response and recovery phase of COVID-19 demand additional nutrient intake; widening the gap of pre-existing undernourishment. Mushrooms, fresh fruits and vegetables, herbs and spices, and legumes are few of such readily available food ingredients which are rich in protein and also have medicinal benefits. BRICS nations have their influences on global development and are highly impacted by a large number of confirmed COVID-19 cases and deaths. The adequacy and access to healthcare are also low in BRICS nations as compared to the rest of the world. Attempt to combat the COVID-19 pandemic are praiseworthy in BRICS nations. However, large population sizes, high prevalence of undernourishment (PoU), and high incidence of mental health ailments in BRICS nations provide a suitable landscape for jeopardy of COVID-19. Therefore, appraising the interplay of nutrition and PNEI modulation especially in BRICS countries will provide better understanding; and will aid in combat COVID-19. It can be suggested that the monitoring will assist in designing adjunctive interventions through medical nutrition therapy and psychopsychiatric management.

## Introduction

The ongoing COVID-19 pandemic affected the various dimensions of well-being in all parts of the globe. As of August 2021, over 216 million diagnosed cases and approximately 4.4 million people have lost their life due to this panic disease worldwide (WHO, 2021). The BRICS countries comprise a wide range of territory cover and substantially share the population, economy, and development at the global level (Jakovljevic, 2014). A significant share in world gross domestic product (GDP) and unique socioeconomic population structure has been linked with essential consideration of BRICS, while designing policies at the global level (Jakovljevic, 2014). These nations are also affected by the severity of COVID-19. The COVID-19 pandemic was originated in one of the member countries of BRICS. Out of the remaining four member countries, three (India, Brazil, and Russia) are among the top five affected countries with the highest number of confirmed cases, along with South Africa in the top 20 (WHO, 2021). Unique socioeconomic, and demographic status of BRICS countries and comparative differences between country members of different economic groups like BRICS, group of seven (G7), emerging seven markets (EM7), and The Organization for Economic Co-operation and Development (OECD) can be linked with their medical preparedness (Jakovljevic, 2014, 2016; Jakovljevic et al., 2020; Carvalho et al., 2021) The rapid increase in affected individuals warrants proactive pharmaceutical solutions and natural remedies to cope up with this ongoing pandemic. The deteriorating effect of COVID-19 is not limited to physical health; it also affected the lifestyle, work culture, and financial welfare (HLPE, 2020; Huang et al., 2020; Soni et al., 2020d). The state of nutrition and neuropsychological well-being has been bilaterally linked with COVID-19 severity. The well-being of physical and psychological health is connected through *P*sycho*n*euro*e*ndocrine*i*mmune (PNEI) system (Soni et al., 2020d; Mehta et al., 2021). Undernourishment has negatively impacted the disease prognosis of COVID-19 (Briguglio et al., 2020; Calder, 2020). On the other hand, pathological manifestations [respiratory distress, gastrointestinal (GI) complications, loss of appetite, and deficient nutrient absorption] caused malnutrition in COVID-19 patients (Zvolensky et al., 2020) Nutritional deficiencies, mainly in protein and vitamin uptake, can have a negative impact on immunity against infections including COVID-19. The unexpected epidemiological burden and post-COVID consequences are disrupting the nutritional status and survival especially those from low- and middle-income countries, and of young age (Rodriguez-Leyva and Pierce, 2021). At the global level, the Committee on World Food Security High Level Panel of Experts on Food Security and Nutrition in its 2020 report discussed the COVID-19 consequences on various dimensions of food availability through initial to long-term effects. In their deliberations, they also pressed the need of considering the complex interaction of nutritional deficiency with outcome including health, society, and psychological well-being (HLPE, 2020). The State of Food Security and Nutrition in the World 2021 report documented a marked escalation in the number of people not having access to adequate food (2.37 billion; 320 million more than that 2019) and facing hunger (811 million; 161 million more than that of 2019; FAO, IFAD, UNICEF, WFP, and WHO, 2021). The prevalence of undernourishment (PoU) among BRICS nations is quite alarming (FAO, IFAD, UNICEF, WFP, and WHO, 2021), India and South Africa have high undernourishment prevalence in their population than the world average. Although. Russia, Brazil, and China have low PoU, these nations share a large undernourished population. Benefits of nutritional supplementation in COVID-19 are speculated as well reported in various investigations (Rodriguez-Leyva and Pierce, 2021). Effector and regulator molecules of psycho-physiological homeostasis are ultimately derived from the diet components. Therefore, the supply of adequate nutrients stands pivotal to fuel the immune system to its optimum as required in abnormal health conditions. Reports indicate that an ample intake of nutrients plays a defensive role against viral infection (Calder, 2020). Elevated consumption of immunity boosters has been reported (Godman et al., 2020; Soni et al., 2021). However, the instability of prices for antimicrobials and vitamins amid the COVID-19 pandemic is a concern to be addressed (Godman et al., 2020). Nevertheless, the hindrance in the immune response can be brought up by common deficiencies and inadequacy of micro- as well as macro-nutrients (Elmadfa and Meyer, 2019; Rodriguez-Leyva and Pierce, 2021). Moreover, bilateral dependency of immunity and psychological status also play a critical role in overall well-being (Soni et al., 2020b).

Neuropsychological consequences and correlation with COVID-19 have surfaced through various reports (Abers et al., 2014; Calder, 2020; García et al., 2020). Nutritional deficiency and exaggerated immune response in COVID-19 patients can invite psychological consequences (Ambrus and Ambrus, 2004; Calder, 2020; Mehta et al., 2021). Moreover, perceived fear, state of uncertainty, and financial crisis amid COVID-19 pandemic effects contribute to such mental distorts. Depressive disorders are quite prevalent in BRICS nations (Ritchie and Roser, 2018).

BRICS nations have a unique state at various global fronts; and hold diversity in economic development, social composition, healthcare practices, food habit, nutritional state, and priority for mental health management (Jakovljevic, 2015, 2016; Jakovljevic et al., 2017; Reshetnikov et al., 2020; Awe et al., 2021; Dash et al., 2021; de Almondes et al., 2021; Popkova et al., 2021; Rababah et al., 2021; Zhu et al., 2021). These nations have distinctive influences of COVID-19 pandemic on society, medical system, and economics (Dash et al., 2021; de Almondes et al., 2021; Rababah et al., 2021; Zhu et al., 2021). BRICS nations are among the countries facing the highest number of confirmed cases of COVID-19 (WHO, 2021) and are affected by associated consequences of malnutrition, and psychological disorders among their population. Members of BRICS have taken measures as a country as well as a group of nations to contribute to combating the COVID-19 pandemic (Reshetnikov et al., 2020; Chattu et al., 2021; Ranabhat et al., 2021). COVID-19 not only affected physical health but also affected mental well-being (Soni et al., 2020d; Mehta et al., 2021). Moreover, the impact of COVID-19 pandemic effects severely affects the production and distribution equilibrium including those of essential items and medicines (Godman et al., 2020; Carvalho et al., 2021; Chattu et al., 2021; Ranabhat et al., 2021). Therefore, deliberations on the association of nutrition, immunity, and neuropsychological state are expected to uncover the potential point in combating COVID-19. As per the unique socioeconomic and healthcare level, the BRICS nations should be especially considered in designing global policies to combat the current pandemic of COVID-19. Existing strategies under practice are advised along with integrated psychiatric interventions (Holmes et al., 2020; Steardo and Verkhratsky, 2020; Tandon, 2020). Nutritional sufficiency through supplementation has been observed to refute the neuropsychiatric altercations in infected patients as well as in post-COVID recuperation. Moreover, nutritional supplements, as well as bioactive components of food items including those rich in nutrition and vested medicinal properties, offer benefits against detrimental manifestations in human diseases including COVID-19 (Singh et al., 2020; Soni et al., 2021). Maintaining the sufficiency of nutrients in affected and susceptible patients will have not only have clinical benefits but can also have preventive advantages (Soni et al., 2021). Moreover, pandemic effects including distorted lifestyle, food habits and stay home measures heavily affect the neuropsychological behavior acting through the PNEI axis. As nutrition is key to maintaining a healthy life and its importance in COVID-19, measures, and guidelines released by authorities at national as well international levels recommend maintaining a good nutritional state and incorporating “immunity boosters” in the daily regimen (Bennett et al., 2020; Khanna et al., 2020; Soni et al., 2020a, 2021). In the time of “COVID-Infodemic,” the overabundance of COVID-related information, the scientific awareness and the spread of reliable information will adjunct the combating approaches. Collectively, it can be suggested that the interplay of nutrition, immunity, and mental health have compounded effects through the PNEI axis; and strategies against pandemic effects must involve these concerns.

## Prevalence in Brics Countries: Drawing an Epidemiological Picture

**Figure 1:**
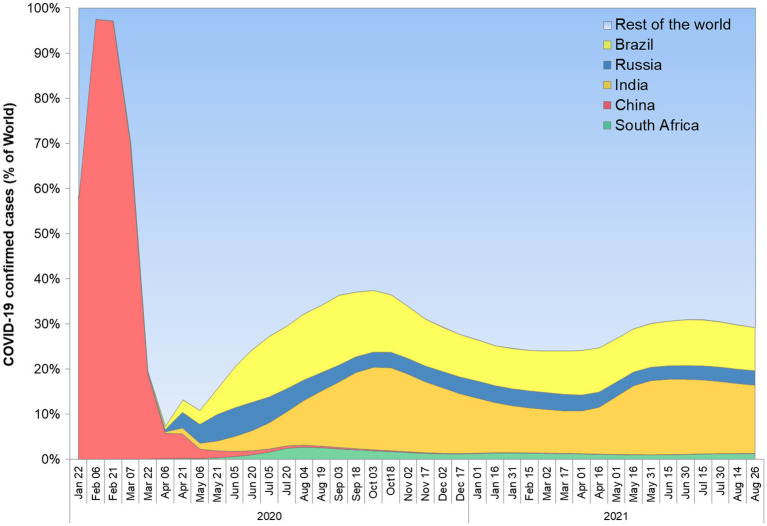
BRICS nations share in confirmed cases worldwide.

BRICS nations share a large fraction of world territory, economics, and population in the world (Godman et al., 2020). They belong to various demographic regions and have diverse ethnicity among their population. The rate of economic growth of these nations surpasses the rest of the world (Jakovljevic, 2014). Further, better forbearance against crisis was reported for Emerging Market Seven (EM7) countries as compared to G7 countries (Jakovljevic et al., 2020). Interestingly, Brazil, China, India, and Russia are common in EM7 and BRICS nations, reflecting the potential of these nations. However, many other factors including climate, culture, and lifestyle affect the transmission and management of infectious health conditions. Despite being among the few largest economies, none of the BRICS nations are in the current member list of organizations for Economic Co-operation and Development (OECD). A comparison of nations from Asia reflects that OECD member countries have better healthcare performance indicators as compared to non-OECD BRICS nations (India and China). The healthcare disparity prevalent in India and China was correlated with heterogeneity among their population at the socioeconomic level (Jakovljevic et al., 2020). Comparing the G7 and BRICS nations, current rate predictions suggest that later groups will surpass in *per capita* growth of the OECD average (Jakovljevic, 2016). However, health disparity, increasing expenses affecting healthcare affordability are among major concerns in BRICS nations (Jakovljevic, 2016). The prevalence of COVID-19 is quite high in these nations. The SARS-CoV-2 was originated in one of the BRICS countries, China, in late 2019, and subsequently affected each part of the globe. The BRICS nations share a large fraction of confirmed COVID-19 cases in the world (Figure 1). As per an estimate, the collective share of BRICS nation COVID-19 cases is approximately 30% (WHO, 2021). Among the top five nations with most cases of COVID-19, BRICS counties India, Brazil, and Russia are standing at 2nd, 3rd, and 4th rank, respectively. South Africa is also among the top 20 nations affected by the most cases of COVID-19.

**Figure 2:**
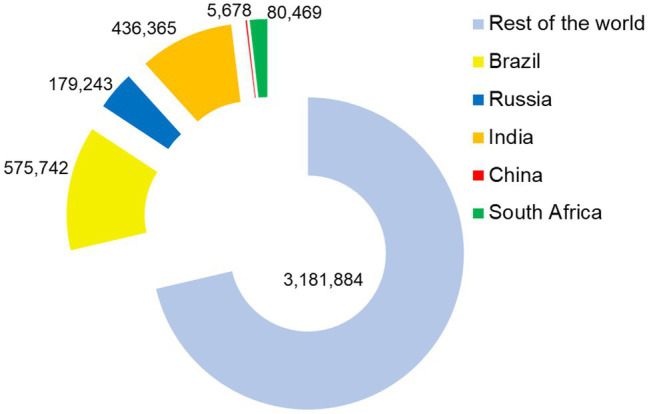
The number of deaths due to COVID-19 in BRICS nations and the rest of the world.

COVID-19 deaths are also high in these nations having Brazil, India, and Russia in the top six affected counties and South Africa among the top 20. About 1.3 million deaths occurred in BRICS nations which accounts for more than 28% of death worldwide (Figure 2). This indicates the severity of COVID-19 in the group of these countries with emerging economies. The healthcare system available in these countries is among the major key factors affecting the COVID-19 outcome. The sufficient number of healthcare individuals, timely diagnosis, and therapeutic interventions along with efficient measures to counter the spread of SARS-CoV-2 infection plays a determinative role in combating the COVID-19 pandemic. The Universal health coverage (UHC) effective coverage index indicates that none of the BRICS nations hold the position among the top 50 countries having good healthcare system (Fullman et al., 2018). China, Russia, and Brazil hold 58th, 63rd, and 74th rank with their UHC effective coverage index (70, 69, and 65, respectively) slightly better than the global average of 60.3. The UHC effective coverage index of South Africa (60) is slightly lower than the global average and ranked 98. Lowest among BRICS nations, India has a UHC effective coverage index of 47, and ranked on 162 among 204 countries assessed. Low access to healthcare will hinder the combat of COVID-19. It will be noteworthy to mention that countries with a good UHC effective coverage index are also impacted by COVID-19; however, the ease and access to healthcare will aid in the management of the COVID-19 pandemic. Through a humanitarian approach, and balancing of social as well as political context the UHC spirit can be achieved in combating the COVID-19 (Jakovljevic, 2015; Ranabhat et al., 2021). Further, the need for intensive promotion of scientific perspective and morality is also pressed to overcome challenges associated with the COVID-19 pandemic (Ranabhat et al., 2021). Moreover, effective utilization and strategic planning can aid the optimal management of COVID-19. As per an estimate, Brazil and South Africa will have a health expenditure, 10.0 and 10.5% of their GDP, comparable with the world average of 10.4% GDP in the year 2025 (Jakovljevic et al., 2017). India’s national health expenditure (<4%) is the lowest among the BRICS nations; however, it is expected to surpass the 4% of GDP in 2025. The projected national health expenditure of Russia (8% of GDP) and China (7% of GDP) are also expected to lag behind the world average (Jakovljevic, 2015; Jakovljevic et al., 2017). The affordability of healthcare and medicine also depends on purchase power parity. However, the privileged vulnerable socioeconomic groups are supported through various government-led schemes for their healthcare (Gauttam et al., 2021). Amid the COVID-19 pandemic, the government of nations including those of the BRICS group offered indiscriminate support to all affected individuals. The insufficiency of the healthcare system has been lessened by public-driven initiatives and volunteer contributions in many countries. Nevertheless, diversity among BRICS nations exists in it is a substantial proportion of the rural population; the public health system is predicted to be effective in nations (Gauttam et al., 2021). Public health expenditure of BRICS countries impacted public health at the world level as well. The promotion of a healthy lifestyle, awareness, and physical activities (including sports activities) can adjunct the other attempts of health maintenance (Jakovljevic et al., 2019). This can be suggested to counter the COVID-19-associated physical and mental health problem.

**Figure 3:**
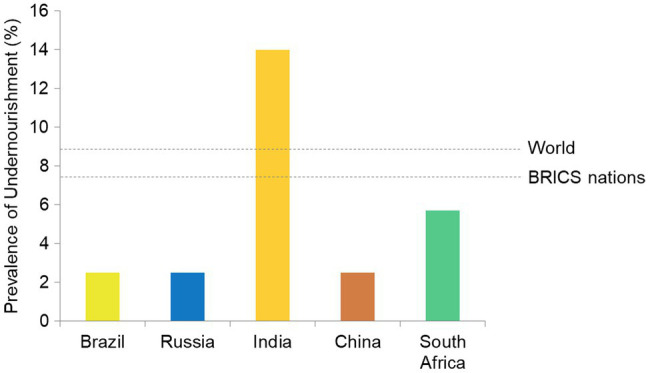
Prevalence of undernourishment (PoU) in BRICS nations.

Among BRICS countries PoU of Brazil, Russia, China (all 2.5), and South Africa (5.7) is low as compared to its global value (8.9; FAO, IFAD, UNICEF, WFP, and WHO, 2021). India has a very high PoU of 14. As PoU is weighted values against population size, these countries share a large number of individuals facing undernourishment. The undernourishment and nutritional deficiencies have wrecking consequences on health, both physically and mentally. The low nourishment state prevalent in BRICS nations (Figure 3) can be suggested as a driving force in COVID-19 morbidities.

**Figure 4:**
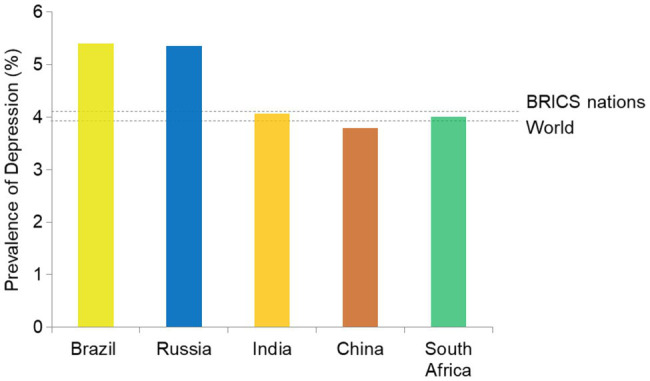
Prevalence of individuals with depression among the population of BRICS nations.

Neuropsychological manifestations were also linked with the manifestations of COVID-19 and their severity. The collective prevalence of depressive disorders in BRICS nations (4.09%) is higher than the global average (3.91%; Figure 4). In absolute numbers, India, China, Brazil, and Russia share the 1st, 2nd, 4th, and 6th highest number of individuals affected with depression. BRICS nations collectively account for approximately 44% global burden of depression-affection individuals. Other mental health disorders also show a similar trend (Ritchie and Roser, 2018).

Owing to performances in nourishment status, healthcare access, mental health level, as well as distribution of population, BRICS nations individually as well as collectively plot a landscape for the menace in COVID-19 pandemic. The BRICS nations applied the measures suggested by WHO and acted promptly to prevent the transmission of COVID-19 through various measures including preventive and diagnostic strategic plans (Reshetnikov et al., 2020; Chattu et al., 2021; Gauttam et al., 2021). The diet habits, lifestyle, level of scientific awareness, and use of immunity boosters/supplements in these regions will have enduring consequences on the COVID-19 pandemic (Roser and Ritchie, 2019). However, the exertions in COVID-19 pandemic management (implementing WHO guidelines, preventive and curative management), and development and use of vaccines against COVID-19 reflect the efforts of these nations (Table 1). To prevent the hindrance in the development and distribution of vaccines, India and South Africa jointly appealed to the world trade organization (WTO) to waive off the trade and intellectual property-related issues to establish global health diplomacy (Chattu et al., 2021). The use of the digital platform for the spread of information and awareness was also observed in BRICS nations. Moreover, digital methods, computation strategies, and artificial intelligence can aid in predictions. Strategies including artificial neural networks have demonstrated effectiveness in predicting the propagation of COVID-19 and vaccination drive outcome in France, Germany, the United Kingdom, Portugal, and Italy (Carvalho et al., 2021). Similar approaches are being utilized in BRICS countries, however, apart from many other factors; the accuracy of data is a major concern.

**Table 1:** COVID-19 vaccine being used in BRICS nations.

Country	Vaccines	Vaccine type	Route	Developer	Manufacturer/Importer	Approval
Brazil	Sputnik V	Adenovirus viral vector, RNA	IM	Gamaleya Research Institute of Epidemiology and Microbiology		Restricted use
	BNT162b1	RNA	IM	Pfizer Inc., BioNTech		Under trial (III)
	BNT162b2	RNA	IM	Pfizer Inc., BioNTech		Approved
	Ad26.COV2.S	Non-replicating viral vector	IM	Johnson & Johnson (Janssen)		Approved
	Covishield	Non-replicating viral vector	IM	Oxford University, Astrazeneca, Serum Institute of India		Approved
	Oxford/Astrazeneca formulation	Non-replicating viral vector	IM	Oxford University + Astrazeneca + Coalition for Epidemic Preparedness Innovations		Approved
	CoronaVac	Inactivated	IM	Sinovac Biotech		Emergency use
	BBIBP-CorV	Inactivated	IM	China National Pharmaceutical Group (Sinopharm) and Beijing Institute of Biological Products		Emergency use
Russia	EpiVacCorona	Protein subunit	IM	Vector Centre of Virology		Approved
	KoviVac	Inactivated	IM	Chumakov Center		Approved
	Sputnik V	Adenovirus viral vector, RNA	IM	Gamaleya Research Institute of Epidemiology and Microbiology		Approved (Emergency)
	Sputnik light	Non-replicating viral vector	IM	Gamaleya Research Institute of Epidemiology and Microbiology		Emergency use
	Convidecia/Ad5-nCoV	Non-replicating viral vector	IM, IN	CanSino Biologics		Under trial (III)
India	Covishield	Non-replicating viral vector	IM	Oxford University, Astrazeneca, Serum Institute of India		Approved
	Covaxin	Inactivated	IM	Bharat Biotech, Indian Council of Medical Research and National Institute of Virology		Approved
	Sputnik V	Adenovirus viral vector, RNA	IM	Gamaleya	Panacea Biotech	Emergency approval
	mRNA-1273 (Spikevax)	RNA	IM	Moderna + NIAID + BARDA	Cipla (filed application)	Emergency approval
	Pfizer	RNA	IM	Pfizer Inc.		Under approval
	Janssen COVID-19	Viral vector	IM	Johnson & Johnson (Janssen)		Emergency approval
	Covovax	Protein-based	IM	Novovax	Serum Institute of India	Under trial (III)
	ZyCoV-D	DNA (Plasmid expressing SARS-CoV-2 protein)	ID	Cadila Healthcare + Biotechnology Industry Research assistance Council (BIRAC)		Awaiting approval
	BBV154	Viral vector	IN	Bharat Biotech + American company Precision virologics + Washington University School of Medicine		Under trial (II)
	Corbevax/BECOV2D/BioE COVID-19	Protein-subunit (Antigen)	IM	Biological E. Limited (BioE), the Baylor College of Medicine in Houston, United States, and American company Dynavax Technologies (DVAX)		Under trial (II)
	HGC019	mRNA	IM	Gennova Biopharmaceuticals and HDT Biotech Corporation with active support from Department of Biotechnology, India		Under trial (I)
China	BBIBP-CorV	Inactivated	IM	China National Pharmaceutical Group (Sinopharm) and Beijing Institute of Biological Products		Approved
	Convidecia/Ad5-nCoV	Non-replicating viral vector	IM, IN	CanSino Biologics		Approved
	SARS-CoV-2 Vaccine (Vero Cells)	Inactivated	IN	Shenzhen Kangtai Biological Products Co. Ltd., and Minhai Biotechnology Co. Ltd.		Approved
	CoronaVac	Inactivated	IM	Sinovac Biotech		Approved
	Inactivated (Vero Cells)	Inactivated	IM	China National Pharmaceutical Group (Sinopharm) and its Wuhan Institute of Biological Products.		Approved
	ZF2001/RBD-Dimer	Protein subunit	IM	Anhui Zhifei Longcom in collaboration with the Institute of Microbiology at the Chinese Academy of Sciences.		Emergency use
	SCB-2019	Protein subunit	IM	Clover Biopharmaceuticals using an adjuvant from Dynavax		Under trial (III)
	INO-4800	DNA	IM	Inovio Pharmaceuticals		Under trial (III)
	BNT162b1	RNA	IM	Pfizer Inc. + BioNTech		Under trial (I)
	BNT162b2	RNA	IM	Pfizer Inc. + BioNTech		Under trial (II)
	Recombinant (Sf9 cell)	Protein subunit	IM	West China Hospital of Sichuan University		Under trial (II)
South Africa	BNT162b1	RNA	IM	Pfizer Inc. + BioNTech		Under trial (III)
	BNT162b2	RNA	IM	Pfizer Inc. + BioNTech		Emergency use
	Ad26.COV2.S	Non-replicating viral vector	IM	Johnson & Johnson (Janssen)		Approved
	Covishield	Non-replicating viral vector	IM	Oxford University, Astrazeneca, Serum Institute of India		Approved
	CoronaVac	Inactivated	IM	Sinovac Biotech		Emergency use

ID, intradermal; IM, intramuscular; and IN, intranasal.

## Viral Manifestations

SARS-CoV-2 infection is associated with complex viral illnesses that range from asymptomatic to severe symptomatic complications. The most critical manifestation is abnormal respiratory health which promotes respiratory disease such as acute respiratory distress syndrome (ARDS), among many other clinical features along with disease progression. The most common manifestations among these clinical features are fever, acidosis, cough, dyspnea, headache, myalgia, nausea, diarrhea, loss of smell and taste, coagulation dysfunction, organ failure, and ultimately death (Guang et al., 2021; Zhu et al., 2021).

About 20% of cases have severe complications such as dyspnea, hypoxia, and >50% lung infection observed in high-resolution-computed tomography (HR-CT) imaging require hospital admission. Among hospital admitted COVID-19 patients, about 5% develop critical disease and need intensive care for the management of hypoxemic respiratory failure or hypotension (CDC, 2021). SARS-CoV-2 severity is also age-dependent, and severity of illness is more frequent in patients of old age as compared to children younger than 10 years (Shi et al., 2020). Nevertheless, many other factors also influence the severity including pre-existing comorbidities. The case fatality rate (CFR) was significantly higher in the older population reported as 14%, whereas lower to approximately 2.3% in general individuals (Amore et al., 2021).

Till now, several general characteristics and diseased elements such as sex, age, high blood pressure, diabetes, chronic lung infection, dermatologic infection, endocrine and hepatobiliary complications, and cardiovascular manifestation are considered among the main risk factor observed during clinical managements of COVID-19 (Jordan et al., 2020; Yang et al., 2020; Jamshidi et al., 2021).

The spectrum of clinical cardiac presentations in COVID-19 patients includes acute coronary syndrome, myocardial injury, cardiac arrhythmias, cardiomyopathy, and cardiac shock, and thromboembolic complications. Some cardiac tests displayed an elevated level of troponin with the more lethal condition of patients (Guo et al., 2020; Shi et al., 2020). Recent reports indicate that immunological manifestations play a potential role in COVID-19 severity *via* promoting the development of extra-pulmonary features (Gupta et al., 2020). These features include thromboembolic complications, arrhythmia, GI symptoms, renal dysfunction, liver dysfunction, diabetic ketosis, and neurological deficits (Gupta et al., 2020; Soni et al., 2020d; Sahu et al., 2021). In addition, some patients can develop an aggressive hyper-inflammatory response, known as cytokine release syndrome, caused by an excessive reaction due to impaired IFN-1 response and transcription factor NF-κB with elevated TNF and IL-6 production (Hadjadj et al., 2020).

Apart from cardiac manifestations, abnormal digestive symptoms were also associated with disease severity and worst outcomes. GI complications include appetite loss [81 (79%) cases], diarrhea [35 (34%) cases], and vomiting [4 (4%) cases] on their clinical data with 204 COVID-19 patients (Pan et al., 2020; Sahu et al., 2021). Therefore, dysregulated GI function became more identifiable as the severity of the disease increased. GI dysregulations also affect the uptake and absorption of nutrients and provoke deficiencies (Sahu et al., 2021). Nutritional defects as a repercussion of anatomical and physiological damages caused by the systemic spread of SARS-CoV-2.

Neurological and psychiatric manifestations are also being observed in many severe COVID-19 patients (Mao et al., 2020). Neuropsychiatric consequences including episodes of anxiety, depression-associated disorders and mood distort were observed in a significantly large fraction of infected patients (Abers et al., 2014; Alpert et al., 2020; Tandon, 2020; Mehta et al., 2021). Notably, clinical data indicated that about 80% of infected cases have a mild infection and/or asymptomatic which can be managed without admission to hospitals with proper monitoring of physicians. However, these asymptomatic patients or those with mild symptoms pose a threat of onward transmission and spread of the pandemic. Nosophobia and uncertainty about treatment outcomes among these patients attract other psychological defects and warrant regular monitoring and counseling. Zubair et al. (2020) indicated that SARS-CoV-2 infection can affect the nervous system *via* several routes such as trans-synaptic transfer across infected neurons, olfactory nerve, infection of vascular endothelium, or leukocyte migration across the blood-brain barrier. According to a recent finding by three neuroscience bodies in the United Kingdom, the neuro-invasion of viruses develop dysphagia after their first acute ischemic stroke in the brain (Aoyagi et al., 2021). The severity of symptoms in COVID-19 patients is also a product of physical and mental health, nourishment, strain of SARS-COV-2, and timely diagnosis of infection.

## Equation of Covid-19 and Malnutrition

Incidence and progression of several illnesses including those of infectious mature largely depend on the nutritional profile of the host and have a key influence on the outcome of therapeutic interventions. The global 2015 famine trend, indicated by the incidence of malnutrition, reversed since decades of consistent declines and has been stabilizing little below ~11% over the preceding 3 years (Ribeiro-Silva et al., 2020). However, a rise in the proportion of individuals suffering from starvation has been observed. As per an estimate one in every nine people across the globe was struggling with hunger in 2018. A recent appraisal in 2020 suggests that a population of 130 million has fallen in this struggle including a large proportion of 44 million minors (HLPE, 2020; Ribeiro-Silva et al., 2020). The socioeconomic distort brought by associated with public heath emergencies cannot be denied to aid in food unavailability to the large section of the population. Even, it is estimated that the current pandemic has the potential to push 49 million people into severe economic distress. Such distresses are forcing the unavailability of nutritive meals, leading to food insecurity and highlighting the enormous difficulty of achieving the Null Hunger objective by 2030 ~ 2050 (Prosekov and Ivanova, 2018; Ribeiro-Silva et al., 2020; UNICEF, 2020). Social restrictions and the potential risk of the contract with COVID-19 also hinder the aids from public initiatives feeding hungry people. These public group-driven initiatives of food donations also dribble due to their impeding financial condition in pandemic-driven economic load. On the communal level, this load decreases productivity, especially food production, and maintains the vicious circle of increased hunger, infection, disease, poverty, and socioeconomic and political instability (Silverio et al., 2021). Hyper infectious nature of SARS-CoV-2, especially it is few variants, countries including BRICS territories have taken unrivaled initiatives to combat COVID-19 transmission, ranging from the limited social exposure, revocation of public transit to social isolation (Zhao et al., 2020; Dash et al., 2021; Popkova et al., 2021; Rababah et al., 2021). Distinct nutrient deficiency or protein-energy deprivation has been shown to detrimentally affect the infection combating prospects along with diminished immunity level (Ambrus and Ambrus, 2004; Silverio et al., 2021).

**Figure 5:**
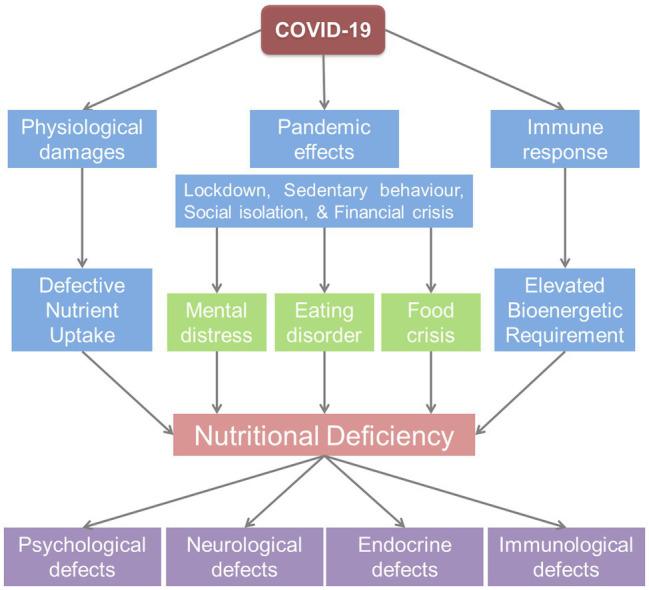
Nutritional deficiency triggered by the COVID-19 pandemic can affect physical and mental health through psychoneuroendocrineimmune (PNEI) modulation.

COVID-19 exhibited a multitude of manifestations, spanning asymptomatic to the severe (Soni et al., 2020a; Silverio et al., 2021). Nutritional deficiencies especially protein-energy malnutrition, body profile, and hyperalimentation (over-nutrition) are among the major risk factors of deterioration, contributing to SARS-CoV-2 response (Cava and Carbone, 2021; Silverio et al., 2021). The risk is accelerated in COVID patients as stirring respiratory illness marked with hypercatabolism and inflammation syndrome, raises energy demands associated with ventilation effort (Thibault et al., 2021). The COVID-19 infection can also affect the assimilation of nutrients as the manifestations include the loss of appetite (Rao et al., 2008). Moreover, COVID-19-driven defects in the expression profile of nutrient-absorbing molecules in the GI tract of patients contribute to nutritional deficiencies (Sahu et al., 2021). These deficiencies culminate in the declined level of neurotransmitters (NTs) essential for mental well-being (França and Lotti, 2017; Amruta et al., 2021). The anxious and depressogenic events in infected patients further strengthen the consequences culminating in malnutrition (Fedotova et al., 2017). Possible routes of COVID-19-associated nutritional deficiencies are illustrated in Figure 5.

Protein Energy Malnutrition is a specific single nutrient deficiency, a frequent etiology of acquired immunodeficiency (SID, Secondary Immuno-Deficiency), and vulnerability to infections (Ambrus and Ambrus, 2004; Ulrich and Kaufmann, 2007). Immune cells have significant calorie consumption and in the events of response such as during infection, these energy and nutrient requirements significantly enhance (Cutrera et al., 2010). An elevation in basic metabolic rate (BMR) observed during fever owing to the activation of the inflammatory response can be attributed to meeting such requirements (Childs et al., 2019). Systemic consequences of lung infection such as cytokine storm and spread of SARS-CoV-2 can invite anatomical damages in multiple organs affecting vital functions. The GI cells (intestinal epithelium) also express ACE2 receptor in abundance, which serves as an anchor for SARS-CoV-2 to infect the host cells. As a result, SARS-CoV-2 infection could impair the digestive function resulting in GI problems (nausea, anorexia, diarrhea, etc.) and a reduction in patients’ nutritional-metabolic status (Huang et al., 2020). Anorexia combined with diarrhea was indicated to add in nutritional disequilibrium resulting in the lag phase of recuperation (Mobarhan and DeMeo, 1995). Therefore, it is clear that SARS-CoV-2 infection will provide a landscape for malnutrition to manifest through direct anatomical and physiological damages leading to deficient absorption of nutrients. Nevertheless, the nutrient requirement in infected patients is higher to meet the demand of responding immune system components (cells and organs). Moreover, the nutrient deprivation and inflammatory consequences in infected patients attract the neuropsychiatric ailments causing stress-driven loss of appetite. The perceived fear of COVID-19 contraction and therapeutic outcome also pour into psychiatric distort in both normal and SARS-CoV-2-infected individuals. The pre-existing malnutrition or deficiencies in specific nutrient have compounded effect with SARS-CoV-2 infection in engaging COVID-19-associated malnutrition and negatively affect the therapeutic outcome. Considering the nutritional deficit and its role in aggravated manifestation of clinical symptoms, food and food supplements able to replenish the calorie requirements can be of immense help. Additional medicinal benefits associated with food ingredients will be of added advantages in many human disorders including COVID-19 (Soni et al., 2020a; Singh et al., 2021). Mushroom is of such ingredients rich in protein content which fulfills energy requirements and also aids in curative as well as preventive measures against various human health anomalies (Singh et al., 2021). The medicinal benefits of mushrooms encompass the antioxidant, anti-inflammatory, anti-infective as well as anticancer activities (Singh et al., 2021).

## The Interplay of Nutrition and Pnei Modulation in Covid-19

**Figure 6:**
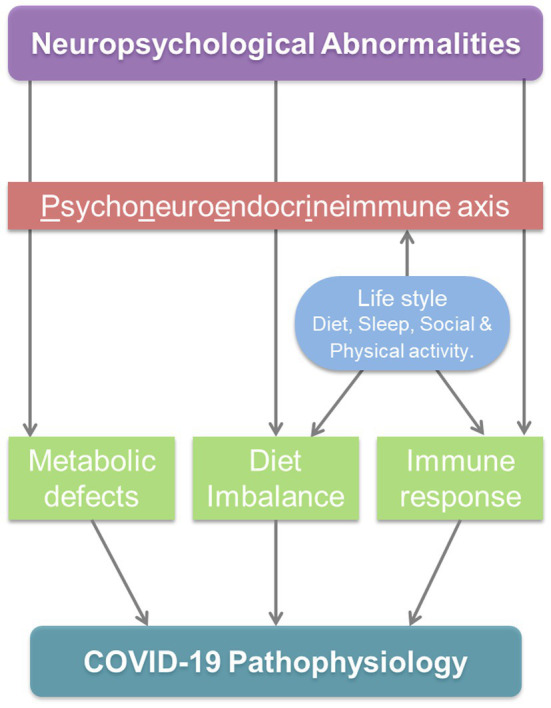
Neuropsychological abnormalities can adjunct the COVID-19 pathophysiology.

The significant loss of muscle mass as well as a deterioration of immunological defenses, which collectively add to the gravity of COVID-19, would thus be linked with the chronic malnutrition driven by SARS-CoV-2 infection (Thibault et al., 2021). Various regulators of immune response and neuroendocrine system (NTs, etc.) are derived from dietary components [amino acids (AAs), etc.]. Moreover, the interdependency of the neuroendocrine and immune system affects both of them even in the circumstances directly deteriorative only for one. Therefore, optimal dietary nutrition is a key to maintain immunity as well mental well-being; undernourishment damages folk’s psychological health, emotional resilience affecting their capability to cherish life and deal with pain, deception, and grief (Ferguson et al., 1999). Intervened regulatory network and interdependency significantly affect the immunological responses under neuropsychological defects. Through PNEI regulatory elements, immunological events/mediators prevailing in the body of the host shake the neuroendocrine processes systemically. The possible triggers of homeostatic imbalance by neuropsychological abnormalities through PNEI modulation are presented in Figure 6. This cross-talk across systems is meticulously pruned by feedback threads that operate in tandem to preserve homeostasis balance (França and Lotti, 2017).

### Metabolic Deficiency as an Induction Loophole

Acute respiratory distress syndrome, PNA inflammation, hyperthermia, consumption coagulopathy (intravascular disseminated coagulation), multi-organ dysfunction, and GI impairment are only a few of the clinical symptoms of COVID-19 (Mitchell, 2020; Soni et al., 2020a; Fedele et al., 2021; Sahu et al., 2021) Among COVID-19 patients, metabolic linkage of psychiatric problems are also invariably mentioned (Mohan et al., 2020; Tandon, 2020). Exposure and risk of contraction with SARS-CoV-2 are majorly governed by lifestyle, presence at public places, and chance incidental acquaintance with infected individuals. However, the establishment of infection and degree of symptom severity is largely affected by nutritional state. Deficiency of nutrients, especially of those which serve as the precursors for immunological or neuroendocrine regulators or mediators, is associated with undesirable disease progression and treatment outcome (Mehta et al., 2021). Therefore, along with attempts to improve the immune response, medical nutrition treatment (MNT) for severely sick COVID-19 patients is being considered against the critical issue of malnutrition-driven complications (Rozga et al., 2021). The absence or deficiency of metabolites deregulates the gradient and availability and creates a shift in homeostasis leading to the manifestation of ill effects. Among many, AAs and vitamins provide crucial mediators of the PNEI regulatory network; and deficiencies of these will negatively affect the strength of infected individuals to withstand mental distorts as well as dampen the immunity level.

#### Amino Acid Deficiency

The deficiency of amino acids has been linked with various pathological conditions. Moreover, the deficiency of this nutrient correlates with severity as well as manifestation of a few exclusive symptoms associated with diseases (Elmadfa and Meyer, 2019; Chen et al., 2020c). COVID-19 is no exception for this. Interestingly, pathological manifestations associated with COVID-19 also pave the path for deficiencies of these critical nutritional components mediating diverse biochemical events. In a systemic spread of SARS-CoV-2 infection, physiological and anatomical damages in the GI tract culminate into impaired absorption of nutrients leads to nutritional deficiency (Sahu et al., 2021). The molecular linkage between SARS-COV-2 infection and amino acid deficiencies is also explored in various investigations. The sodium-dependent neutral amino-acid transporters (*B^0^AT1*) expression level is suppressed by the aberrant form of SARS-CoV-2 entry gate enzyme, angiotensin-converting enzyme-2 (ACE-2; Warner et al., 2020). The diminished expression of nutrient transporters including *B^0^AT1* impairs the absorption of AAs (Huang et al., 2020; Wang et al., 2021). The resultant imbalance in the amino acid pool leads to diverse pathophysiological consequences both on physical and mental health (Carnegie et al., 2020). The expression of flawed *B^0^AT1* in COVID-19 patients is correlated with psychiatric signs similar to those in Hartnup disorder, which also shares defective expression of amino acid transporters (Soni et al., 2020d; Mehta et al., 2021). Moreover, anorexia in COVID-19 also contributes to the deficiencies of nutrients including amino acids. Anorexia is induced by secondary clinical manifestations in COVID-19 like GI issues; and physiological impairment triggered by viral infection impedes nutrition intake, resulting in nutrient mal-absorption. Pre-existing amino acid deficiencies are also speculated to enforce the severity of ill effects associated with COVID-19 (Bedock et al., 2020; Rozga et al., 2021). Through potentiation of anti-infective immune response, dietary nutrients keep immune response optimal to prevent establishment of any invading pathogen in the host body. Although contrasting links were observed between malnutrition and clinical signs associated with SARS-CoV-2 infection, studies found a positive correlation between poor prognosis, severity, and mortality with deprived nutritional state (Nicolau et al., 2021). Early nutritional profiling of infected patients is suggested to favor better clinical outcomes through supplement or diet management (Bedock et al., 2020). Though nourishment therapy delivers necessary nutrients, it is feasible that supplementing regular nutrition support with conditional amino acids may result in a greater capacity for recuperation and nutritional stability (Rozga et al., 2021).

Amino acids are not just the architectural element of proteins, they are also essential in the homeostasis of emotional and neuropsychiatric wellness. Disturbances *via* dietary shortfalls cause mood swings as they serve as precursors for various NTs and hormones regulating the psychological consequences and its interrelation with PNEI (Slominski et al., 2002; Mehta et al., 2021). Such detrimental consequences aid up in the deteriorative effects of COVID-19 (Zubair et al., 2020). The biosynthesis of 5-hydroxytryptamine, 5-HT (Serotonin) requires Tryptophan, an essential AA; and the deficiency of tryptophan lowers the serum concentration of serotonin (Bjork et al., 2000). Pioneer investigations have revealed that the lower levels of tryptophan in plasma are linked to a greater likelihood of aggression, anxiety, and depressive disorders (Bjork et al., 2000; Von Polier et al., 2014). Psychological resilience and neuroticism are dictated by metabolic derivatives of amino acids (including tryptophan catabolites; TRYCAT). Numerous NTs including GABA, dopamine (DA), epinephrine, and norepinephrine are deduced from a diverse spectrum of amino acids (including glycine, tyrosine, phenylalanine, glutamate, etc.; Rao et al., 2008; Carnegie et al., 2020; Mehta et al., 2021). Amino acid anomalies in COVID-19 are anticipated to influence both physiological and mental well-being. Supplementation of conditional amino acid and/or incorporating food items rich in protein/amino acid contents can be expected to not only overcome the bioenergetics deficiencies but will also provide benefits in maintaining the psychosomatic homeostasis.

#### Vitamin Paucity

Vitamins have their role in preventing infection through strengthening the anti-infective immunity. Since the onset of the pandemic, the immuno-nutritive potency of vitamins, especially Vitamin C and D, has gained much attention. Accumulating findings have begun to give greater weight to possible links between the prevalence and severity of COVID-19 with vitamin levels of the infected patient (Lanham-New et al., 2020; Lordan, 2021). Vitamin D has also been shown to affect the pathological consequences of many other diseases. In obese persons with relation to geography and age, the prevalence of vitamin D (Vit D or calciferol) inadequacy is 35 times greater (Holick, 2017). Vit D, a fat-soluble secosteroid hormone, can act as an immunomodulator as well as antioxidant (Lee et al., 2018; Lanham-New et al., 2020). Moreover, it has historically been linked with altered hematopoiesis and PNEI reactions to optimize immunity and cognitive health (Fedele et al., 2021; Mehta et al., 2021).

Vitamin D enhances the synthesis of respiratory epithelial antimicrobial peptides (such as human β-defensin-2 and cathelicidin) and therefore reduces the frequency of disease onset and COVID-19 effects (Mitchell, 2020; Hernández et al., 2021). As the twin response, it is also engaged in the prophylaxis of viral respiratory tract infections (RTIs) and acute lung damage, as documented in ARDS condition, where lung permeability diminishes due to modification of ACE-2 expression and renin-angiotensin system interaction (Mitchell, 2020; Fedele et al., 2021). Calciferol deficiency has also been aligned to neuropsychiatric afflictions such as Autism and major depressive disorder (MDD). Furthermore, in COVID-19, a decreased serum 25-hydroxyvitamin D level has been related to psychological distress signs (Basheer et al., 2017; Lordan, 2021). The levels of serotonin (5-HT), DA, and estradiol (E2) in the brain are all affected by vitamin D deficiency as it activates neuronal activity by triggering vitamin D receptors (VDR) in the central nervous system (Di Nicola et al., 2020; Mehta et al., 2021).

Other players of the vitamin bandwagon, like Vitamin A, B, and E, are also playing a significant role in combatting COVID-19 infection and their deficiency may too result in crisis. Vitamin A and B are shown to be critical for maintaining gut integrity; and deficiency will lead to impairment in barrier function (Yoshii et al., 2019; Calder, 2020). In asymptomatic individuals without chronic pulmonary problems, vitamin A depletion is linked to a minimal forced vital capacity FVC (FVC). A low FVC is an indicator of airway blockage and a substantial marker of fatality. Functional maturation of phagocytes neutrophils and macrophages are also affected by Vitamin A depletion leading to impaired ability of these cells to kill pathogens (Calder, 2020). Among the vitamin B family, Vitamin B1, B3, and B5 are majorly involved in the regulation of phagocytosis and inflammatory cytokine production by macrophages (Yoshii et al., 2019). As B3 can resist the production of inflammatory cytokine (Lipszyc et al., 2013) and favors the differentiation of regulatory cells through GPR109a signaling (Lipszyc et al., 2013; Yoshii et al., 2019), its deficiency is suggested to exaggerate the inflammatory consequences and cytokine storm in COVID-19. Along with innate immune responses, Vitamin D also supports acquired immunity by driving the proliferation and functionality of B and T lymphocytes. Similarly, vitamin C also protects cells from oxidative stress as well as acts as an immune system regulator suppressing the release of pro-inflammatory cytokines (Elmadfa and Meyer, 2019). According to a recent comprehensive analysis, intravenous (IV) vitamin C treatment might minimize the need for mechanized ventilation presumably by curbing lung damage, without influencing the overall risk of death (Zhang and Jativa, 2018).

Vitamin A deficiency can negatively affect the optimal formation of specific antibodies after immunization attempts (Penkert et al., 2019). Moreover, vitamin D deficiency deteriorates the seroprotection after vaccination against respiratory viral disease (Lee et al., 2018). Deficiencies of different vitamins are prevalent in BRICS countries (Awe et al., 2021), and are anticipated to hinder the optimum effectiveness of COVID-19 vaccination drives in these parts of the globe. Various publications have advocated for the supplementation of vitamins in SARS-COV-2 infected patients as well as to the individuals receiving COVID-19 vaccines for better recovery and immune response (Calder, 2020; Shakoor et al., 2020).

Despite the role in immune response modulation, vitamins also affect the psychoneurological inter-regulation (Soni et al., 2020b; Mehta et al., 2021). Nevertheless, vitamin deficiency-induced defects in immune response also affect psychosomatic well-being through PNEI modulation (Cornish and Mehl-Madrona, 2008; Calder, 2020; Soni et al., 2020b). The positive correlation between the severity of COVID-19 symptoms and undesirable clinical outcome with vitamin deficiencies (Mohan et al., 2020) can be suggested to have a contribution in frequent psychiatric manifestations in infected patients. Moreover, the restriction measures at the social and occupational level along with uncertainty about health, perceived fear, and associated factors act as additional factors contributing to deficiencies of these psycho-immune active vitamins. The most notable vitamin in the linkage of COVID-19 and depressive disorders is vitamin D (Shakoor et al., 2020; Ceolin et al., 2021). Vitamin D plays a critical role in the maintenance of the chronobiological rhythms through Vitamin D binding protein and VDR (Jones et al., 2017; Ceolin et al., 2021). Vitamin D also acts as an antidepressant; upholds serotonergic neurotransmission through induction of tryptophan hydroxylase 2 gene expression (Ceolin et al., 2021).

Unique prevalence status of psycho-neurological disorders in parts of BRICS countries (Charlson et al., 2016) and deficiencies of nutrients (Awe et al., 2021) can be suggested to have a causal association. These deficiency frequencies in BRICS countries will have additional contributions in defective immune response and associated neuropsychological consequences in the COVID-19 pandemic dissimilar to other parts of the world. The paucity of medicines including nutritional supplements and vitamins in parts of the world including BRICS nations affect the optimal management of the COVID-19 pandemic (Jakovljevic, 2015; Godman et al., 2020). Although countries of the BRICS group have taken quite similar measures to prevent the spread of SARS-CoV-2, the preparedness and attempts to prevent psychological ailments seem to be different (de Almondes et al., 2021).

### Role of Altered Immune Response

In many contexts, malnutrition has been associated with immunological dysfunction, notably hunger and cachexia. It has been confirmed with investigations both in human and animal models. Nevertheless, starvation also hampers T cell cytokine synthesis along with significantly decreasing T cell counts (Mengheri et al., 1992). A cumulative rise in the generation of pro-inflammatory cytokines and an infantile inflammatory reaction is attributed to an elevation in fat saturated with N-6/N-3 PUFAs (Teasdale et al., 2019). The systemic energy equilibrium including glucose and lipid metabolism is governed by a variety of PUFAs operating as natural ligands for PPARs and SREBP (Muralikumar et al., 2017). Consequently, an imbalanced n-6/n-3 PUFA ratio in serious mental illness (SMI) patients may add to hyperglycemia, dyslipidemia, and obesity risk indirectly *via* PPAR and SREBP transcriptional activation. This theory may be relevant to COVID-19 since persons with such conditions have a higher risk of catastrophic consequences (Choi et al., 2020; Zhou et al., 2020).

The investigators hypothesized a link between ingesting foods with high anti-ACE activity and having a low COVID-19 mortality rate (Nadalin et al., 2021). Few of the countries with low mortality rates such as Bulgaria, Greece, and Turkey record for consumption of fermented milk, which is known to be an organic ACE inhibitor. These observations might be explained by a decrease in angiotensin II (Ang II) synthesis, and preventing the pro-inflammatory state and subsequent acute lung damage leading to more drastic consequences COVID-19 embodiment (Warner et al., 2020). According to research from China, individuals with progressed COVID-19 had remarkably accelerated inflammatory indices in their bloodstream, and plasma ferritin, C-reactive protein, and IL-6 levels were considerably enhanced in non-survivors than veterans (Chen et al., 2020b; Zhou et al., 2020).

Further, bioenergetics requirements during immune responses in COVID-19 are heightened leading to utilization of available nutrient resources. It will be noteworthy to mention here that owing to social distancing measures and restricted outdoor activities, the nutritional imbalance prevails in large geographical areas of the world including those of BRICS countries (Zhu et al., 2021). This is linked with health vulnerability in the region with the incidence rate of infections (Zhu et al., 2021). The activation of immune response and associated energy demand can cumulatively shore up the nutrient deprivation worsening the PNEI response. The severity of COVID-19 symptoms and cytokine storm has been linked with incidences of depression episodes in patients. Molecular events of inflammatory immune responses like the stimulation of NLRP3 inflammasome have been linked with neuro-invasion and onset of neuropsychiatric disorders (Ribeiro et al., 2021). Moreover, anxiety about treatment outcome along with other depressive disorders leads to loss of appetite. Depression itself has been shown as an inducer of an inflammatory immune response. Low food intake and mal-absorption can expectedly hamper the optimal immune response as well as neuropsychological well-being.

### Dietary Restriction as a Triggering Agent

Diseases triggered by nutritional imbalance, as well as other dietary ailments, have been considerably reduced in populations in developed countries. This is owing to an upgraded insight of the priority of nourishment, augmentation of specific food items, and rapid substantial improvements in the standard of living (Childs et al., 2019). However, dietary restrictions are prevalent in parts of the globe, which can be suggested to be an underlying cause of nutritional imbalance. The factors influencing the diet restrictions range from non-availability of specific food items, preferential choice due to customary dietary habits and religious beliefs (Persynaki et al., 2017). These dietary restrictions practiced among diverse socioeconomic and religious groups have both beneficial and harmful health consequences (Persynaki et al., 2017; Pourabbasi et al., 2021). Such dietary restrictions may lead to nutritional insufficiency that attracts various health conditions. The diverse religious groups and climatic conditions in BRICS countries affect the diet practices and can be suggested to influence the COVID-19 consequences. Although socio-scientific links can be traced to the climate and agricultural practices in the region of origin for the specific religion or practice, a customary adaptation of such food habits/restrictions exists (Tan et al., 2013). The copious presence of groups practicing fasting, for a specific time or food items, can be seen in the territories of BRICS countries. Preventive measures associated with the COVID-19 pandemic also differentially influenced the dietary habits of various groups of the population (Bennett et al., 2020). Moreover, food allergies and intolerance compel absentees from certain food items (HLPE, 2020). The pandemic also affected the availability of safe food items to these allergic patients which further increases the risk of deficiencies (Mack et al., 2020). Psychological manifestation linked with a pandemic is also suggested flaring the food allergic response in individuals. Nutritional inadequacies can bring up temporal immunodeficiencies and have neuropsychological consequences (Sarris et al., 2015). Dietary restrictions-led deficiencies in either physical or mental health will be prompting the defect in others through mediators of the PNEI axis (Bennett et al., 2020; Mehta et al., 2021). It will be noteworthy to mention that scientifically planned diet restrictions have health benefits even in COVID-19 (Ambrus and Ambrus, 2004; Rozga et al., 2021; Silverio et al., 2021). Recently gaining a diverse form of vegetarianism including vegan, lacto-vegetarian diets, etc., may lead to deficiencies of some nutritional elements. Such specific diet habit-driven deficiencies can be replenished with conditional supplementation (Soni et al., 2020b; Mehta et al., 2021; Rozga et al., 2021). Many of the COVID-19 patients under recuperation have preferential food habits due to lifestyle and religious practices, and these supplementations may aid the convalescence in these patients. One of the sought strategies to supplement the essentials is probiotics (Mack et al., 2020; Mak et al., 2020). While probiotics supplementation has demonstrated direct association with the immune system and may avoid many kinds of infections, delusional use of standard probiotics for combating COVID-19 is not advised until understanding its influence on the intestinal microbiota and SARS-CoV-2 pathogenesis (Mak et al., 2020). The optimal dietary approach in the management of COVID-19 patients relies on the individual’s health status (Childs et al., 2019; Rozga et al., 2021; Sahu et al., 2021). For the patients with COVID-19 consumption issues, whey protein fortification and a supplement that combines vitamins and minerals covering daily requirements are suggested (Hernández et al., 2021). Conversely, regimens with meals of various textures and consistence, readily digested (e.g., yogurt or custard, fruit mousse, fruit slices, and soft cheese), of at least 25–30 kcal/day, are advised for individuals that are not in a severe condition (Yamamoto et al., 2020). The general method for handling COVID-19 people from the ICU to the clinical ward is comprised of dietary interventions, medical nourishment treatment, careful supervision, and prompt follow-up. Optimal nutritional interventions, particularly in the fragile aged, immuno-compromised, and those with dysthymia who may be undernourished or at the nutrition risk can assure longevity along with a healthier and faster recuperation from this condition (Barazzoni et al., 2020). The dietary restriction-associated neuroimmunological consequences can be averted by surveillance and information management of nutritional status and dietary monitoring.

### Influences of Activity Deprivation

COVID-19 pandemic affected the various dimensions of life. The measures taken to counter the rapid spread of SARS-CoV-2 distorted the lifestyle and work practices (Soni et al., 2020b). The lockdown and work-form-home adaptations restricted the outdoor activities. This distorts put an unprecedented and unwelcomed but compelling alteration in daily routine culminating into changes in diurnal activities (Hammami et al., 2020; Kumar and Nayar, 2021). These deviations in routine physical activity, outdoor movements, and social interactions create a depressogenic state conducive for medium and long-term psychological consequences (Holmes et al., 2020). Stay at home condition is an oddity that compels individuals to change regular lives and everyday routines. Behavioral adaptations to these activity-restricted conditions in ongoing pandemic hamper the desire to do feasible work (Girdhar et al., 2020). This confining situation triggers mental pathologies of psychosomatic origins like anxiety issues, frustration, shifts of circadian rhythm, insomnia, impulsiveness, hypervigilance, etc (Clemente-Suárez et al., 2020; Kumar and Nayar, 2021). Episodes of PTSD and major depression disorder (MDD) have been observed to increase with the periods of isolation and confinement (Soni et al., 2020b). Among many, age has been a critical factor affecting the manifestations of these psychiatric conditions (Soni et al., 2020d; Steardo and Verkhratsky, 2020). Physical activity restrictions have detrimental consequences on the qualitative and quantitative magnitude of immune response including those in COVID-19 (Hammami et al., 2020). The onset of psychiatric disorders can also be suggested as a maneuvering force for wrecked immunity (Clemente-Suárez et al., 2020). Through the modulation in the PNEI network, fluctuations in the immune system, psychological state, and neuroendocrine coordination are conveyed to each component (França and Lotti, 2017; Soni et al., 2020b; Steardo and Verkhratsky, 2020). Social distancing measures have a detrimental influence on physical activity and had prolonged the hours of sitting and lying (Hammami et al., 2020). Moreover, COVID-19 patients with prolonged lying on bed and artificial ventilation usually incur deep muscular weakness, decubitus ulceration (bedsores), dysautonomia, and respiratory dysfunction (Yamamoto et al., 2020). These conditions evoke a stringent pulmonary illness balancing abnormalities, post-intubation regurgitation, postural hypotension, deep venal thrombosis (DVT), joint contractures, etc. (Yamamoto et al., 2020), and thus, need extra care and rehabilitation. Given the necessity of physical fitness maintenance, concepts of home-based workout with physical performance assessments tailored as in-house fitness alternatives are propagated for the amid the COVID-19 outbreak (Chen et al., 2020a; Clemente-Suárez et al., 2020). Suggestive performance checks on daily basis and application of practical residence practices can offset the deleterious repercussions of the passive lifestyle during solitary confinement (Chen et al., 2020a; Clemente-Suárez et al., 2020; Hammami et al., 2020). Alleviating the physical health through these modalities during COVID-19 pandemic can also be suggested to improve the digestion, nutrient balance, and immunity along with improved neuropsychological health.

### Perceived Nosophobia and Psychiatric Link

COVID-19-related neuropsychiatric problems, including depression, anxiety, traumatic stress disorder, and so on, tend to be prevalent, and cover a broad variety of fractious phenotypes that have a detrimental influence on the quality of life (Ribeiro et al., 2021). Social distancing measures, isolation, and quarantine, and uncertainty are among plausible reasons for the discontent observed in service users seeking psychiatric treatment for personal well-being and emotional resilience (Barazzoni et al., 2020; Tandon, 2020). Unfortunately, this epidemic has led to a rise in maladaptive survival strategies such as the use of materials as well as suicide (Czeisler et al., 2020; Amruta et al., 2021). In the last couple of years, the involvement of the immune interface with the CNS framework in psychiatric health and stress response has been the focus of extensive investigations (Calder, 2020; Aoyagi et al., 2021), which uncovered many conventions facilitating neuropsychiatric comorbidities by immune stimulation (i.e., by viral infection; Steardo and Verkhratsky, 2020).

Symptoms of PTSD are being observed in SARS-CoV-2 infected patients as well as among those who have not contracted the COVID-19 (Lai et al., 2020; Mao et al., 2020; Aoyagi et al., 2021). Nosophobia of COVID-19 and uncertainty of subsequent treatment outcomes have also contributed to these observed neuropsychiatric consequences (França and Lotti, 2017; Soni et al., 2020d; Steardo and Verkhratsky, 2020; Mehta et al., 2021). Bilateral contributions from immune stimulation and psychological distress through PNEI regulation also exist (França and Lotti, 2017; Steardo and Verkhratsky, 2020; Mehta et al., 2021). Prolonged distress boosts the level of pro-inflammatory chemokines like TNF-α, interleukins (IL-1, IL-6) and stimulates activation of indolamine-2,3-dioxygenase (IDO). IDO triggers the generation of tryptophan catabolites (TRYCATs) which are psychoactive. Under the homeostatic state, the levels of neurotoxic and neuroprotective TRYCATs are balanced; and any imbalance can be sought to elicit PNEI disturbances (França and Lotti, 2017; Steardo and Verkhratsky, 2020). In the pandemic, TRYCATs have established a health degradation anxiety-immune-neuropsychiatric-immune cycle (Soni et al., 2020b). The patients admitted to the intensive care unit (ICU) are frequently stressed; feel mental health burden frequently observed in severely affected by COVID-19 (Yamamoto et al., 2020). In addition to infected patients, medical professionals working at the forefront also have fairly high emotional and physical strain and develop signs of anxiety, sadness, sleeplessness, and discomfort in their working environment, particularly in high-risk regions (Lai et al., 2020; Yamamoto et al., 2020). Long working hour demand in management of pandemic restricts their dietary routines. The inadequacy of medical professionals in parts of globe including those in BRICS countries also contributes to demand of long working hours without any resting periods. This can be suggested as underlying factor driving the physical, psychological, and nutritional stress. These stresses make healthcare professional more susceptible in their work environment. Therefore, it can be suggested that prevailing dietary restrictions are affecting the COVID-19 risk and severity; and the contractions of infection and measures to combat pandemic may also drive the constraint on diet practices. The monitoring and maintaining the diet as per guidelines and medical conditions can have an adjunct effect in alleviating the ill effects of on-going pandemic.

## Therapeutic Measures and Remedial Plan

Until recently, there is no proven and effective antiviral treatment exists to mitigate the COVID-19 infection. The current treatment guidelines and management strategies vary between countries worldwide. The inclusion and weightage on the management of psychological manifestation and their influences through nutrition and immune response also varies in different countries. The bidirectional cooperation between COVID-19 and psychiatric disorders is being reported (Wang et al., 2020). The PNEI mediators are also suggested to deteriorate the immune responses through this linkage of COVID-19: psychiatric consequences. Owing to the crucial role of nutrition in both immune response and mental health, dietary management holds a good place in the therapeutic regimen against COVID-19. Therefore, the recommendation of one approved guideline at a global scale might be highly appreciated in all aspects of treatment planning. However, the rapidly growing evidence in SARS-CoV-2 research provides a significant number of sites for potential drug targeting. Currently, some conditional solutions and psychological canceling are available for the clinical management of the ongoing viral outbreak. In the current scenario, the vaccination program is on the high priority to break the chain of infection to prevent further transmission of COVID-19.

### Conditional Treatment

To date, there is no specific and effective antiviral treatment established to fight COVID-19. Although there are many antiviral drugs under initial investigation and clinical trial phase which may hold promise against coronavirus-2.^1^ Repurposing clinically evaluated old drugs are the major available therapeutic candidates that come from established clinical pieces of evidence from the previous pandemic such as SARS-CoV, MERS-CoV, and other influenza virus outbreak (Ratre et al., 2020). These conditional options are the only available cure for identification, mitigation, and deployment of treatment of COVID-19-associated pathological manifestations. Toward this end, some previously approved antiviral therapies including the HIV-1 protease inhibitors lopinavir (LPV) and ritonavir, the hepatitis C protease inhibitor danoprevir, and the influenza virus RNA-dependent RNA polymerase inhibitor (RdRp) favipiravir are under clinical investigation (Cao et al., 2020).

Lopinavir is a clinically tested agent, against HIV-1 and influenza viruses, approved for combinational treatment with fix dose of ritonavir (Chu et al., 2004). A review of studies reveals that investigations observing the effectiveness of combination therapy of lopinavir/ritonavir are clinical case reports, small retrospective; nonrandomized cohort studies (Yao et al., 2020). This makes it more challenging to ascertain the straightforward benefits of these potential agents. Nevertheless, a recent randomized clinical trial of lopinavir/ritonavir (400/100 mg, twice-daily for 14 days) in 199 hospitalized patients with severe COVID-19 and found no significant effect with lopinavir/ritonavir treatment compared with standard therapy (Cao et al., 2020). In addition, Deng et al. (2020) reported that combination therapy of LPV-r and arbidol was associated with improved lung morphology observed by pulmonary computed tomography images. Favipiravir is an RdRp inhibitor that blocks the replication of a virus *via* acting as a prodrug of a purine nucleotide (Sanders et al., 2020). This agent has significant antiviral activity (EC_50_ = 61.88 μM/L) against SARS-CoV-2 (Wang et al., 2020). However, the limited number of clinical data is supporting the use of favipiravir for COVID-19. A randomized clinical trial-based study with 120 moderate and severe SARC-CoV-2 patients demonstrated the administration of favipiravir compared with Arbidol and found that there is no clinically significant difference in recovery at day 7 with both drugs (71.4% favipiravir and 55.9% Arbidol, *p* = 0.019). Therefore, further investigation of favipiravir is recommended for the treatment of COVID-19 (Chen et al., 2020d). Ribavirin, another antiviral agent has been considered as a hopeful therapy for the management of COVID-19 owing to its effectiveness against previously existing SARS-CoVs. Wang et al. (2020) demonstrated its extensive *in vitro* potential against COVID-19 (strain WIV04). In addition, a retrospective study in the city of Wuhan, China with 134 clinically approved severe COVID-19 patients was conducted (Tong et al., 2020). However, due to unfavorable reactions pattern, the proper dose of ribavirin in the clinical application should be standardized; and be given with proper monitoring according to the patient’s severity. US-FDA granted the emergency use of remdesivir in the second wave of COVID-19. Remdesivir, a viral RNA polymerase inhibitor; and in investigations including a clinical trial, it showed significant antiviral activity (EC_50_ = 0.77 μM; EC_90_ = 1.76 μM) against several coronaviruses including novel COVID-19 (Wang et al., 2020; Kalil et al., 2021). Therefore, this agent is a highly recommended therapy for COVID-19 at the early stage of infection (Al-Tawfiq et al., 2020; Wang et al., 2020). More recently, a cocktail of remdesivir with Baricitinib was found to significantly reduce the recovery time and drive improved outcomes in those COVID-19 patients receiving high-flow oxygen or noninvasive ventilation (Kalil et al., 2021). However, remdesivir is only available as an intravenous fluid (IVF), and the effectively of the drug is yet to be established for critically ill ICU patients. Other FDA approved antiviral remedies such as penciclovir, Oseltamivir, etc. are under experimental phase for possible antiretroviral management of COVID-19.

Besides drugs with established antiviral activity, other agents 19 are also considered for their effectiveness against COVID-19. Among these, chloroquine and hydroxychloroquine have a long-standing history in the prevention and treatment of malaria and autoimmune diseases (Savarino et al., 2003). However, chloroquine has been previously reported to possess broad-spectrum antiviral effects (Savarino et al., 2003; Gao et al., 2020). Interestingly, it was previously known for its potent inhibitory action against SARS-CoV *via* blocking the ACE2 receptor (Savarino et al., 2003; Warner et al., 2020). Furthermore, clinical shreds of evidence demonstrated that the COVID-19 virus enters the epithelial cells of mucosa *via* ACE2 receptor, and chloroquine can act at both entry and post-entry levels of COVID-19 infection (Gao et al., 2020; Wang et al., 2020). One recent study depicted that hydroxychloroquine is more effective and acts even at lower concentrations compared to chloroquine against SARC-CoV-2. Further, its immunomodulatory action has also been noted that could synergistically accelerate the antiviral potential *in vivo* (Yao et al., 2020). During previous virus outbreaks including the SARS pandemic, interferons were extensively used as treatment measures. IFN-*β* inhibits the *in vitro* replication of viruses including SARS-CoV (Hensley et al., 2004). Therefore, IFN-*β* was proposed as a choice of candidate for COVID-19 treatment. One of the studies found that Type I IFN significantly declines the viral protein load and replication of SARS-CoV-2 (Lokugamage et al., 2020). However, further supportive pieces of evidence are required to support this therapeutic regime. Monoclonal antibody therapies are also in the line of investigation and clinical trial phase for the treatment of COVID-19. However, few of them are also in clinical practice based on identified benefits from investigations with small number of patients (Xu et al., 2020; Yao et al., 2020). Tocilizumab, an IL-6 receptor antagonist, is FDA approved to treat rheumatoid arthritis and cytokine release syndrome following chimeric antigen receptor T-cell therapy. Due to these past therapeutic history, tocilizumab has been used in patients with severe COVID-19 with early reports of success (Xu et al., 2020). In COVID-19, psychological distress along with neurological disorders accompany the pathophysiological manifestations and correlates with the severity of the disease. Although symptomatic treatment of physical health deterioration lessens the associated mental health consequences, therapeutic interventions may directly affect the neuropsychological magnitudes in patients (García et al., 2020). Steroidal anti-inflammatory agents, through steroid receptors with their abundant presence in the hippocampus, affect the psychological well-being of patients even in COVID-19. Psychological effects of corticosteroids in COVID-19 range from mild to moderate including hypomania, depression, and mood disorders (García et al., 2020). Neurotoxicity of antiviral drugs is also reported (Abers et al., 2014). Moreover, lopinavir/ritonavir induced the loss of taste (Abers et al., 2014) may lead to low appetite and consequently poor nutrition. Delirium-like symptoms with usages of antibiotics prescribed in therapeutic regimen (Sirois, 2002) can be expected to negatively affect the nutrition and immune response. Such consequences will have a compounding effect and hinder the objectives of treatment. Further, IFN-*β* therapy has been linked with severe depression disorders (Fragoso et al., 2010). Interestingly, tocilizumab have the protective effect against psychotic disorders and can be speculated to improve the mental health along with the physical heath of COVID-19. Although, direct-acting action on mental health is not well established for most of the therapeutic agents used for COVID-19, their ability to modulate PNEI response can largely affect the recuperation phase. Therefore, its neuropsychiatric consequences and patients’ mental health must be taken into account during designing therapeutic regimens at a personalized level.

Medical nutritional therapy is also taken into consideration for clinical management of COVID-19 (Bedock et al., 2020; Calder, 2020; Rodriguez-Leyva and Pierce, 2021; Silverio et al., 2021; Thibault et al., 2021). The food ingredients including those rich in calories are being considered to improve the nutritional status of the patients affected with COVID-19 (Barazzoni et al., 2020; Bedock et al., 2020; Calder, 2020; Fedele et al., 2021; Nicolau et al., 2021; Thibault et al., 2021). Protein-rich food items such as mushrooms and legumes can provide benefits to various human disorders (Rodriguez-Leyva and Pierce, 2021; Silverio et al., 2021; Singh et al., 2021). Moreover, the medicinal properties of food ingredients like mushrooms, herbs, and spices also aid in clinical measures to combat COVID-19. Conditional amino acid therapy replenishes the essential amino acids in affected patients (Soni et al., 2020d; Mehta et al., 2021; Rozga et al., 2021). Food items enriched with essential amino acids such as mushrooms, legumes, seafood can also fulfill the requirement, at least partially, and prevent the requirement of conditional amino acid therapy. The integration of the “dietetics approach” in the management of COVID-19 will have adjuvant benefits to the clinical measures against this pandemic.

### Herbal Remedies

Historically, the traditional medicinal plants have been known as universal healers. Historical uses for their medicinal properties, easy availability, minimal side effects, and low cost make them potential candidates against diverse forms of human pathologies. Natural herbs are an extensive source of antiviral recipes and immune boosters. Currently, compounds of herbal origin are playing a potential role in the disease prevention and cure against several clinical illnesses including the ongoing COVID-19 (Goothy et al., 2020; Khanna et al., 2020; Soni et al., 2020a). There is a wide range of herbal drugs used in traditional Chinese medicine or Ayurvedic medicinal practices, which were extensively explored against previous CoV outbreaks including *Astragali Radix* (*Huangqi*), *Saposhnikoviae Radix* (*Fangfeng*), *Glycyrrhizae Radix Et Rhizoma* (*Gancio*), *Atractylodis Macrocephalae Rhizoma* (*Baizhu*), and *Lonicerae Japonicae Flo* (Luo et al., 2020; Soni et al., 2021); and are expected to aid in the clinical management of on-going pandemic. However, the lack of adequate data and inconsistent results on herbal remedies against COVID-19 necessitate further studies to understand antiviral mechanisms at the molecular level (Wang et al., 2020).

Metabolites of some traditional herbal medicine are known to possess modulatory activity on ACE2 (Khanna et al., 2020; Soni et al., 2020a; Warner et al., 2020). This includes curcumin, tanshinones, magnolol, baicalin, withanone, tinocordiside, and rosmarinic acid (Khanna et al., 2020; Shree et al., 2020; Saggam et al., 2021). Further explorations of these natural herbs and their components against COVID-19 are required. Notably, curcumin is highly suggestive as a gold standard remedial option for the cure of COVID-19 infection in many literature reviews. Curcumin is a broadly explored agent hijacking the several steps of viral biochemistry by acting as a protease inhibitor, cellular signaling pathways modulator (Soni et al., 2020c; Zahedipour et al., 2020). Moreover, traditional plant-derived metabolites are recently characterized as modulators of immune responses including cytokines and eicosanoids levels (Khanna et al., 2020). In some preclinical experiments, crude extract of *Sambucus nigra* L., *Echinacea angustifolia* DC. and *Echinacea purpurea* L. (Moench.), Larch (*Larix* sp.) and plant extracts or food supplements rich in Vitamin D increases the production of IL-1β and IL-18 by immune-deficient cells (Alschuler et al., 2020). Authority on practices of alternative and complementary medicine systems, AYUSH (Government of India) advised the use of herbal medicines for the management of COVID-19. Among these herbs, tulsi (holy basil) is highly suggested for SARC-CoV-2 (Goothy et al., 2020). Additionally, AYUSH experts recommended some remedial plans to cope up with this current outbreak including the use of *Tinospora cardiofolia* (Giloy) for chronic fever, *Andrographis paniculata* (Kalmegh) for fever and cold, *Cydonia oblonga* (Quince), *Zizyphus jujube* (red date), *Withania somnifera* (Ashwagandha) for cold and flu, and *Cordia myxa* (Assyrian plum) are recommended as antioxidant, immune-modulator, anti-allergic, smooth muscle relaxant, anti-influenza activity (Goothy et al., 2020; Khanna et al., 2020; Shree et al., 2020; Saggam et al., 2021). Homeopathic medicine *Arsenicum album* 30 also acts as an immune-modulator and found effective against SARS-CoV-2 infection (Maurya et al., 2020). For the management of respiratory manifestation associated with COVID-19, Ayurvedic preparation including Agastya Haritaki and Anu taila (oil) are recommended (Vellingiri et al., 2020).

The direct antiviral effects of herbal preparations along with immunomodulatory potential have been sought as exigent benefits in COVID-19 (Shree et al., 2020; Saggam et al., 2021). Apart from these, neuropsychological benefits are also suggested (Aubry et al., 2019; Khanna et al., 2020; Soni et al., 2020b). Many of these bioactive phytochemicals alleviate psychosomatic stress (Fedotova et al., 2017; Aubry et al., 2019; Soni et al., 2020b). COVID-19 pandemic effects have distress consequences (Clemente-Suárez et al., 2020; Popkova et al., 2021; Rababah et al., 2021); and herbal medicinal preparation or bioactive phytochemical-rich diet can moderate these social distress disorders (Aubry et al., 2019). Due to uncertainty on COVID-19 pandemic, anxiety and similar disorders become frequent. The phytochemical has been shown to annul a wide range of these disorders (Fedotova et al., 2017). Herbal drugs can be expected to deliver benefits either directly or through indirect action on components of the PNEI system. Alleviating neuropsychiatric distress will improve the immune response and recuperative ability of patients. Although traditional medicinal preparations are being used based on their established benefits against symptomatic manifestations, many of them are also under early-stage validation for their efficacy in the clinical management of COVID-19 (Maurya et al., 2020).

### Psychological Counseling

In addition to the deterioration of physical health, the psychological burden during the ongoing pandemic dramatically influences the treatment outcome and recovery of COVID-19 patients affecting millions of lives worldwide (Clemente-Suárez et al., 2020). The psychological reaction may vary from aggressive or panic behavior to pervasive feelings of hopelessness and desperation (Thakur and Jain, 2020). These reactions connect with negative psychological extremes including suicidal behavior (Thakur and Jain, 2020). From the onset of the viral outbreak in 2019 to current counter measures including lockdowns, quarantines, and other restrictions are enforced infrequently compelling people to stay home and work-from-home (Brooks et al., 2020). Therefore, individuals are experiencing deviations from their normal behavioral patterns and report anxiety, depression, lack of concentration, and mood disorders (Rao et al., 2008). In these vents, psychology/psychiatry associations and authorities recommend counseling to mitigate the burden of lockdown and quarantine in the COVID-19 situation on mental health (Kontoangelos et al., 2020). The need for psychological counseling is to enhance mental well-being through bringing modulation in decision making and behavioral changes during a time of crisis (Hammami et al., 2020; Lai et al., 2020). Globally this has been accompanied by the implementation of various public health policies and guidelines designed to fix routine life style through alleviation of psychological distress and establishment of mental and physical well-being (Brooks et al., 2020). Emerging pieces of evidence depict the psychological impact of COVID-19 on individuals directly as well as indirectly in both infected and healthy individuals including the near ones of infected patients and those who are facing social distancing measures (Alschuler et al., 2020; Brooks et al., 2020; Guang et al., 2021). Indeed, it should be emphasized that the majority of the population is not expected to suffer from mental illness forced from the pandemic and its impact (Taylor, 2019). Recently, a study conducted in India identified the key stressors acting as potent drivers for bad psychological illness including fear of one’s health, the well-being of the family, economic difficulties, sense of isolation due to quarantine, job security, disturbed social systems and overabundance of misinformation (infodemics) by media and other sources (Banerjee, 2020). These facts indicate that abnormal psychological nature is damaging the people’s lives during COVID-19 pandemic. Therefore, demonstration of the benefits of psychological science for mental well-being through scientific strategies is warranted. This will also influence the socio-economic status of the society (Clemente-Suárez et al., 2020) and will provide adjunct benefits in COVID-19 pandemic.

### Mental Health Welfare and Scientific Awareness

COVID-19 is one of the major global health crises at present which hurt millions of people by influencing their lives in several ways including physically, socially, psychologically, and spiritually. It is now well documented that the COVID-19 pandemic poses a major burden to mental health (Loades et al., 2020; Vindegaard and Benros, 2020; da Silva et al., 2021). Psychologically, COVID-19 pandemic effects act as major drivers of stress impacting mental fitness worldwide. The pre-existing mental disorder can negatively affect the immune response and prompt the severity of COVID-19. A large number of reports indicate dramatic disruption of mental health and behavior with SARS-CoV-2 infection (Cunningham and Firozi, 2020). Notably, the mental health hotline in the United States experienced 10 times increase during pandemic-associated lockdown (Raw et al., 2021). Notably, the parts of the world with a low economy have compelling priorities to improve their physical healthcare system over mental healthcare. The inadequacy of mental healthcare cannot be negated in compounding the detrimental consequences amid COVID-19 (Antonova et al., 2021). Some medical data reported elevated abnormal mental nature and more suicides probably because of poor mental fitness (Hollyfield, 2020). Therefore, to address mental health welfare and scientific awareness a broad range of government policies and assignments are highly recommended to win the battle in eradicating the COVID-19 pandemic. First of all, there should be a priority to identify the common psychological threats and myths associated with COVID-19. In addition, various mental awareness programs should be run by the healthcare system to reduce the pandemic induced fear, stress, and depression. Scientific awareness among the population about COVID-19 is expected to aid in efficient combating strategies. Government authorities and public initiatives are also contributing to spreading awareness about COVID-19. The existence of scientific awareness is high in a few investigations (Singh et al., 2020). However, the existence of a huge amount of information on various sources creates an Infodemic”; and causes a state of confusion among common people in the decision to follow which advice. The data mining, processing, and artificial intelligence methods can be expected to assist in designing combat strategies and forecasting their success (Carvalho et al., 2021). Nevertheless, among many other factors, practices of convenience sampling due to pandemic measures may also deviate the finding from the true prevailing scenario. Therefore, continuous efforts to spread awareness about the disease, preventive measures, and related information must be ongoing.

## Discussion

The interplay of nutrition and the PNEI system is very crucial for manifestations of disease and its course. The COVID-19 pandemic has a large impact not only on human health conditions but also impacted human lives through disturbing the social and economic conditions and interactions at local as well as at global levels (Godman et al., 2020; Carvalho et al., 2021; Chattu et al., 2021; Soni et al., 2021). The impact of COVID-19 is also relayed to human lives through distorting of daily routines and leisure activities (Alschuler et al., 2020; Godman et al., 2020; Soni et al., 2021). The differences in countries belonging to different economic groups like BRICS, G7, EM7, OECD, etc. at demographic and socioeconomic levels served as a factor in combat against COVID-19. Although the COVID-19 affected each part of the world, the countries’ priority toward the healthcare sector, their preparedness, the difference in policymaking, and strategies for their executions served as a decisive factor in recuperation from this pandemic. The differences at the demographic and socioeconomic level in the population residing in BRICS nations and country members of other economic groups like OECD, G7, and EM7 also determined the pace of COVID-19 spread, the effectiveness of containment strategies, and clinical measures. The preexisting differences in healthcare systems in different country members of various groups including BRICS cannot be neglected to have a major role in the effective management of COVID-19. The guidelines recommended by various regulatory organizations including WHO have been implemented by most of the countries; however, the local restraints also exist. The religion and lifestyle practiced in countries, even in some parts, have a large impact on the success of pandemic measures both at the social level and at the clinical level. The differences in food habits among populations belonging to different countries, regions, religions, and believes is also a precursor of the nutritional status of individuals and the population at large. The food preferences and restrictions are also derived from religious beliefs and have an impact on nutritional balance. Restrictions in food items and intake can be permanent observance or can be observed during special religious days at weeks, months, or annual cycles. The diurnal restrictions in food habits can also affect the susceptibility of individuals toward many infectious disorders including COVID-19. Apart from socioeconomic differences, the nourishment status of different countries’ populations also exists. Most of the BRICS nations, except India, have a smaller population fraction suffering from undernourishment as compared to the world level (Figure 3). However, the large population size of India affects the collective nourishment status of BRICS nations. Psychopsychiatric wellness has also been shown to affect the susceptibility toward disease conditions including SARS-CoV-2 infection as well as therapeutic measures. The prevalence of mental illness is higher in most of the BRICS nations compared to its world average (Figure 4). As per the recent statistics, only China has the lowest prevalence of depression in the BRICS countries which is also smaller than the world average. However, in many regions of the world including those in BRICS nations, mental illness is not given priority and medical consultation is not a common measure leading to underreporting. Interdependence of nutrition and mental well-being suggests the coexistence of nutritional deficiency and neuropsychiatric illness.

The COVID-19 drives the nutritional deficiency through raising energy and nutrient demand as well as through physiological and anatomical damages caused in the GI tract. While the preexisting nutritional deficiencies can worsen the outcome, owing to PNEI modulation, the neuroendocrine disturbances compound the manifestation of the disease in COVID-19 and lead to severe symptoms, systemic spread and associated morbidities. The large proportion of the population affected with either nutritional deficits, mental illness, or both in BRICS nations have the high risk of undesired outcome of SARS-COV-2 infection and measures for its clinical management. Moreover, the steps to prevent the spread of SARS-CoV-2 infection including lockdown, and social distancing led to financial uncertainty and hindrance in the availability of essential supplies including food items and medicine (Godman et al., 2020; Chattu et al., 2021). Although guidelines are being released at the national and global level by the regulatory organization, the absolute implementation is mostly far from achieved due to demographic, socioeconomic, population size, religious beliefs, educational status, and healthcare availability reasons. The country members of other economic groups are also impacted with COVID-19 and have undergone a sharp surge in confirmed COVID-19 cases. However, strategies against COVID-19 and their probability of implementation with success differ due to differences exist among members of BRICS nations as well as G7, EM7, and OECD members. Among many factors influencing COVID-19 manifestations, availability of healthcare (UHC effective coverage index) and preexisting physical and mental health conditions among the population can be expected as the major determinant for the initial surge of confirmed cases of COVID-19 and deaths. As the evidence from investigations start pouring, the medical councils/authorities of individual nations as well WHO provided the standard operating procedure for SARS-CoV-2-infected patients. Initial investigations were primarily focused on the molecular pathology of SARS-CoV-2 infection; however, later investigations demonstrated the involvement of nutritional deficiencies and neuropsychiatric illnesses in worsening the COVID-19 pathological manifestations. Moreover, the impact of social measures to prevent spread of SARS-CoV-2 infection drive the psychological derailment; and is also suggested to be considered for effective management of the COVID-19 pandemic (Girdhar et al., 2020; Soni et al., 2020b,d; Kumar and Nayar, 2021; Mehta et al., 2021; Nadalin et al., 2021).

The measures have been taken by member countries from different economic groups against COVID-19; however, the collective measures by BRICS nations will have a huge impact on global outcome owing to large contribution in worldwide population, economy, and terrestrial area. BRICS nations have developed or are manufacturers of a major proportion of vaccines against COVID-19 (Table 1). Along with the modern medicine system, traditional medicinal practices are common in BRICS nations including Ayurveda, Traditional Chinese Medicine, Russian Traditional medicine, African traditional medicine, and Brazilian Traditional Medicine. Most of these traditional medicinal practices use herbs as their preferential ingredients to prevent and cure various human diseases. Ayurveda and other traditional medicine practices also recommend specific diet preferences during different human illnesses. It reflects that nutritional management during disease conditions is already an integrative part of traditional medicine practiced in countries including members of BRICS. Along with modern medicine, integration of traditional medicinal practices has also been recommended by WHO to boost the immunity and prevent he contract with infectious agents including SARS-CoV-2.

As the experiment-based evidence reflects the involvement of nutritional management and PNEI as one of the major determinants of COVID-19 manifestation, most of the national and global authorities recommend the incorporation of these courses of actions in standard operating procedures. However, the existing prevalence of mental illness and undernourishment in BRICS nations and other similar countries must be included in consideration in designing the policies to combat the current pandemic. Although, the distortion in daily routine including decreased physical activity and financial uncertainty brings anxiety and depression along with nutritional imbalance impacting every country, the unique status of each member country of the BRICS group must be considered critically in the investigation of the consequences of COVID-19. This will also help in designing successful strategies at the global level to combat the COVID-19 pandemic not only at the clinical level but also socioeconomic front.

## Conclusion

**Figure 7:**
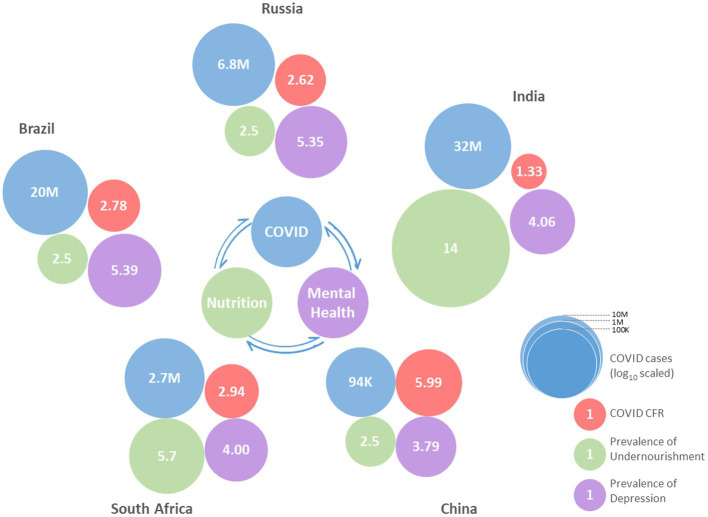
Set-up of COVID-19 cases, case fatality rate (CFR), undernourishment, and mental disorders in BRICS nations.

Nutrition and neuropsychological disorders play a critical role in the causes and consequences of COVID-19 severity. The interplay between the PNEI axis and nutrition largely affects the outcome of therapeutic interventions in COVID-19. The high confirmed cases and COVID-19 death number in BRICS nations reflect the perilous condition. The nutritional deficiencies may aggravate the COVID-19 severity, and high undernourishment (PoU index) among BRICS countries poses a threat of severe SORS-CoV-2 infections in their populations. The nutritional state has an accumulative effect on physical as well as mental health through PNEI modulation. The qualitative and quantitative immune responses are at least partially governed by the psychological state of the individuals. The bilateral regulatory events between neuroendocrine and immune response are superintended by nutrition-derived level and type of nutrition-derived factors. The high prevalence of mental health disorders in BRICS nations is here suggested as a factor having compounded effect on protection and immunity against COVID-19. The country members of other economic groups have a distinctive state as compared to BRICS nations. Many factors affect the COVID-19-associated manifestations including clinical symptoms and socioeconomic consequences. Amid the COVID-19 pandemic, the perceived fear attracted the neuropsychiatric presentations; and financial and economic losses lead the increased food insecurity causing an escalation in undernourishment globally as well as in BRICS nations. Collectively this sets a favorable stage for COVID-19 spread and comorbidities (Figure 7). Various strategies have been suggested to prevent this interdependent “tsunami” of psychological consequences and COVID-19. Conditional supplementations (amino acids and vitamins) can be suggested to improve both immunity and mental well-being. Considering the prevalence of undernourishment and mental health conditions in BRICS and a large number of COVID-19 cases, the nutritional monitoring and psychological interventions especially for COVID-19 affected patients can be suggested to have adjunctive influence in the clinical management of SARS-CoV-2 infection.

## Author Contributions

NV, DS, and SP conceptualized the study. AM, YR, VS, KS, and AT contributed to literature search and analysis. Critical evaluations and revisions were made by NV, DS, SK, and KS. All authors contributed to the article and approved the submitted version.

## Conflict of Interest

The authors declare that the research was conducted in the absence of any commercial or financial relationships that could be construed as a potential conflict of interest.

## Publisher’s Note

All claims expressed in this article are solely those of the authors and do not necessarily represent those of their affiliated organizations, or those of the publisher, the editors and the reviewers. Any product that may be evaluated in this article, or claim that may be made by its manufacturer, is not guaranteed or endorsed by the publisher.

## Footnotes

^1^ https://clinicaltrials.gov
